# Riparian *Ficus* Tree Communities: The Distribution and Abundance of Riparian Fig Trees in Northern Thailand

**DOI:** 10.1371/journal.pone.0108945

**Published:** 2014-10-13

**Authors:** Pornwiwan Pothasin, Stephen G. Compton, Prasit Wangpakapattanawong

**Affiliations:** 1 Department of Biology, Faculty of Science, Chiang Mai University, Chiang Mai, Thailand; 2 School of Biology, University of Leeds, Leeds, United Kindgom; 3 Department of Zoology and Entomology, Rhodes University, Grahamstown, South Africa; 4 World Agroforestry Center c/o Knowledge Support Center for the Greater Mekong Sub-region, Faculty of Social Sciences, Chiang Mai University, Chiang Mai, Thailand; University of Delhi, India

## Abstract

Fig trees (*Ficus*) are often ecologically significant keystone species because they sustain populations of the many seed-dispersing animals that feed on their fruits. They are prominent components of riparian zones where they may also contribute to bank stability as well as supporting associated animals. The diversity and distributions of riparian fig trees in deciduous and evergreen forests in Chiang Mai Province, Northern Thailand were investigated in 2010–2012. To record the diversity and abundance of riparian fig trees, we (1) calculated stem density, species richness, and diversity indices in 20×50 m randomly selected quadrats along four streams and (2) measured the distances of individual trees from four streams to determine if species exhibit distinct distribution patterns within riparian zones. A total of 1169 individuals (from *c.* 4 ha) were recorded in the quadrats, representing 33 *Ficus* species (13 monoecious and 20 dioecious) from six sub-genera and about 70% of all the species recorded from northern Thailand. All 33 species had at least some stems in close proximity to the streams, but they varied in their typical proximity, with *F. squamosa* Roxb. and *F. ischnopoda* Miq the most strictly stream-side species. The riparian forests in Northern Thailand support a rich diversity and high density of *Ficus* species and our results emphasise the importance of fig tree within the broader priorities of riparian area conservation. Plans to maintain or restore properly functioning riparian forests need to take into account their significance.

## Introduction

*Ficus* (Moraceae) is one of the largest plant genera, with more than 750 described species distributed worldwide, mainly in tropical countries [1]–[3]. Fig trees occupy a diversity of habitats and display a wide range of growth forms that includes hemiepiphytes (strangling figs and banyans) and large woody climbers as well as trees and shrubs. The positions of their fruits (figs) include cauliflory (inflorescences borne on the trunk), on the surface of the soil and among the leaves, making them available to a wide range of frugivores [4]. Fig trees are often ecologically important ‘Keystone’ components of tropical forests, because of the large number of vertebrates that feed on their figs, more than on any other group of plants [5]. They are often abundant and are the most abundant genus in soil seed banks in tropical Asia [6], [7]. Fig trees are especially prominent in the riparian zones within SE Asian forests [8], [9], though only a small number of species are extreme riverine habitat specialists capable of growing on rocks in mid-stream (rheophytes). Other species are bank-side specialists that are largely restricted to riparian zones, and others are more widespread species, also found routinely in other habitats [10].

The specific identification of *Ficus* species can be difficult, but fig trees as a group are relatively easy to recognize. They are defined by their figs (syconia), unique enclosed inflorescences lined with tens to thousands of tiny, usually unisexual flowers [2], [11]. *Ficus* species may be monoecious or functionally dioecious. Monoecious figs produce figs containing male flowers and female flowers with various style lengths. All trees have the same type of figs, which can produce wasps, pollen and seeds. Gynodioecious figs are functionally dioecious and produce male and female figs on separate trees [12]. Male figs contain male and short-styled female flowers [13]. The male flowers provide pollen and the female flowers nourish developing wasps, which develop in galled ovules. Male figs rarely produce seeds [14]. In contrast, female figs, which contain only long styled female flowers, exclusively produce seeds. The female flowers can be pollinated, but their long styles prevent pollinating wasps from depositing eggs into ovules [12], [14]. Most fig trees are pollinated by females of one or a small number of host-specific species of fig wasp (Agaonidae) and have an obligate mutualistic relationship with them [15]–[22].

At least 47 *Ficus* species are native to northern Thailand [23]. Some fig trees are early successional pioneers that have been used successfully as framework species in regenerating a degraded upper watershed of evergreen and seasonal forest in Doi Suthep-Pui National Park in northern Thailand [24]. In this ‘framework species’ restoration method about 20 percent of planted seedlings are recommended to be fig tree species [24]. Their value for restoration stems from the birds that are attracted to feed in fig trees, which then also disperse seeds of other tree species, thus adding to tree species diversity in rehabilitated forest areas [25]. As food resources, the all-year-round production of figs among monoecious species, often in large quantities, can potentially maintain vertebrate frugivore populations at times when other fruits are in short supply and this continuous fruiting may be a mechanism that helps maintains biodiversity in tropical rain forests [5], [26]–[29]. Hence, the diversity and reproductive phenology of *Ficus* have special relevance for conservation and tropical forest ecosystem integrity.

Riparian zones are important interfaces (ecotones or transition zones) between streams and surrounding terrestrial habitats [30]. Natural riparian zones are among the most diverse, dynamic and complex terrestrial habitats in the world, but they are very sensitive to environmental change [30], [31]. Streamside vegetation has an important function in river and stream ecosystems, as it supplies allochtonous organic matter (including leaf litter and terrestrial invertebrates), filters the nutrients and pollutants that reach the streams, helps stabilize banks, and influences water temperatures and light through shading [30], [32]–[38]. There is often variation in plant species composition closer to streams [39], and maintenance and conservation of riparian areas therefore contributes to the diversity of the entire forest landscape [40].

Environmental disturbance to riparian forests, caused by factors such as road building, clearance and other human activities, is increasing, and has had a direct impact on native biodiversity [41]. Extensive agricultural and urban development and associated soil erosion have contributed to the decline of riparian forests in Thailand, but the significance of this decline is poorly documented. There are few estimates of how many plant species are found exclusively in riparian zones, but *Ficus* is believed to be the most diverse genus in riparian forests of Thailand [42], [43]. We studied the species richness, diversity and distribution relative to stream edges of the fig trees growing in riparian zones in Northern Thailand, with the objective of improving understanding of the distribution limits and habitat requirements of this ecologically important group of species. Our objective was to answer the following questions: (1) Is there a distinct group of *Ficus* species associated with the riparian zones? And (2) Are there gradients in community metrics such as stem density or species richness as a function of distance from the streams? We hypothesized that in this forest landscape, riparian zones may provide a sufficiently different abiotic or environmental template to result in exclusively riparian tree species or community characteristics.

## Methods

### Study Site

Our study area was located in the Ping River basin in Northern Thailand (18° 51′ N, 98° 54′ E) (Fig. S1), which covers an area of about 35,000 km^2^. It comprises an upper tributary sub-basin of the Ping River, which in turn is the largest tributary of central Thailand’s Chao Phraya River. The region is mountainous with some lowlands along river tributaries, with about 60 percent of the land area having an elevation above 500 m. The climate of Northern Thailand is characterized by three distinct seasons, the rainy, the cool dry and the hot dry seasons. Mean monthly temperatures for a 25-year period (1988–2012) at the Chiang Mai meteorological station varied from 14°c in January to 36°c in April. The average annual rainfall, relative humidity, and annual temperature were 1,072.5 mm, 70%, and 25.9°c, respectively [44]. The basin is subjected to the southwest monsoon during May to October and to tropical cyclonic storms from the South China Sea at the end of the rainy season between September and October. The average annual rainfall and runoff from the basin are around 1174 mm and 6815 million m^3^, respectively [45]. The Ping River basin comprises tertiary continental basin-fill sediments underlain by older Paleozoic gneissic granites, Paleozoic sediments and volcanics and Mesozoic granitic rocks [46].

Stream sections of orders two to four were monitored along four perennial streams (each at least 1000 m long) during October 2010–December 2012. The stream sites were located in hill-evergreen, mixed-deciduous and deciduous-dipterocarp forests [47]–[49] (Table 1). They mostly have granite and gneiss as the dominant rock types and all contained large boulders, were bedrock-lined and carried only small amounts of silt and sand. The streams flow all year, with rapid temporary increases in flows in the rainy season (June–October). Stream MKL is distinct. It is larger than the other streams, has the highest elevation and it is partly fed by a small natural hot spring. This stream also has a limestone substrate and flows through hill evergreen forest, deciduous dipterocarp forest and agricultural land before joining the Ping River.

**Table 1:** Geological and environmental characteristics of the study sites.

Study Site	Elevation(m asl.)	Substrate	Forest type(Maxwell & Elliot 2001)	Streamwidth (m)
Huay Kaew stream (HK)	472−1680	granite	deciduous dipterocarp, mixed deciduous, and evergreen forests	5.0−12.0
Mae Klang stream (MKL)	450−2000	granite	deciduous dipterocarp, mixed deciduous, and evergreen forests	7.0−20.0
Mae Sa stream (MS)	690−1490	granite	deciduous dipterocarp, mixed deciduous, and evergreen forests	7.0−18.0
Mae Ka stream (MK)	490−527	granite and limestone	deciduous dipterocarp, and mixed deciduous forests	3.5−5.0

### Species Diversity

The abundance of each *Ficus* species was estimated using the belt transect method [50]. Ten 20 m wide belt transects that extended 50 m from the stream edge were set at each site. A stratified random sampling design was employed, to cover all the elevation ranges and forest types in each study site. All *Ficus* species present in the transects were identified following *Flora Malesiana* and *Flora of Thailand* [10], [51].

For each belt transect, *Ficus* species richness was taken as the total number of fig species present (all growth forms). Species diversity in each site was calculated using the Shannon-Weaver diversity index (H′) which accounts for both abundance and evenness [52], in the package PRIMER [53]. The Shannon index ranges typically from 1.5 to 3.5 and rarely reaches 4.5 [54]. Other parameters such as species evenness (e) and Simpson’s diversity index (1-D) were also derived [55].

Descriptive statistics were computed to compare the mean abundance, species richness, diversity indices and evenness values of fig trees at the four study sites. All variables were compared using one-way analysis of variance (one-way ANOVA), after verifying normality and homogeneity of variances. Tukey HSD tests were used when ANOVA resulted in significant differences [56].

### Fig Tree Distributions

To reveal individual species distributions in relation to streams, we measured the distance of all individual trunks to the stream edge using a tape measure and then generated a median distance of each fig tree species from the stream. In addition to calculating total stem density versus distance from streams, each belt transect (20×50 m) was also divided into four contiguous 5×50 m subplots. Species richness and number of stems were calculated in each subplot. A two-way ANOVA tested how species richness and stem abundance differed between streams and in distance bands from the streams (0–5 m, ≥5−10 m, ≥10−15 m and ≥15−20 m). Linear regressions determined the relationship between mean species richness and number of stems in each distance band by using the minimum distance to the stream of each subplot. All tests were applied using R version 3.0.0 [56].

### Fig Tree Composition Along Elevation Gradients

We classified all quadrats (n = 40) from four streams according to their elevations. The elevation gradient was divided into three ranges: 400–600 m asl (n = 16), 600–1,000 m asl (n = 11) and >1,000 m asl. (n = 13) (Fig. S2). These ranges followed the forest classification schemes in Thailand; dry deciduous dipterocarp, mixed deciduous and evergreen, and hill evergreen respectively [47], [49], [57]. One-way ANOVA then compared the mean abundance, species richness, diversity indices and evenness values, and density (stems/1000 m^2^) between the three elevation ranges. Normality and post hoc tests were conducted as before [56].

We used linear regressions to determine the relationship between number of stems, species richness, species diversity (H′) and elevation by using the mean elevation of each quadrat. We generated similarity matrics (Sorensen’s index) of species abundance per quadrat and all regressions using the Labdsv and Vegan packages in R. We performed Fuzzy Set Ordination (FSO) analysis on the similarity matrics. The FSO analysis creates an axis that is relativized (zero to one) using both a vector variable and the similarity matrix [58]. In this study, the environmental vector variable was the mean elevation of each quadrat. The resulting relativized axis is termed the “apparent elevation”, based on the similarity matrix of species abundance, and mean elevation of the quadrat. In this way, the environmental vector of “elevation” was used to predict the species composition of the quadrats, and their variation in relation to the environmental variable, in this case, elevation.

## Results

### Species Richness

**Figure 1:**
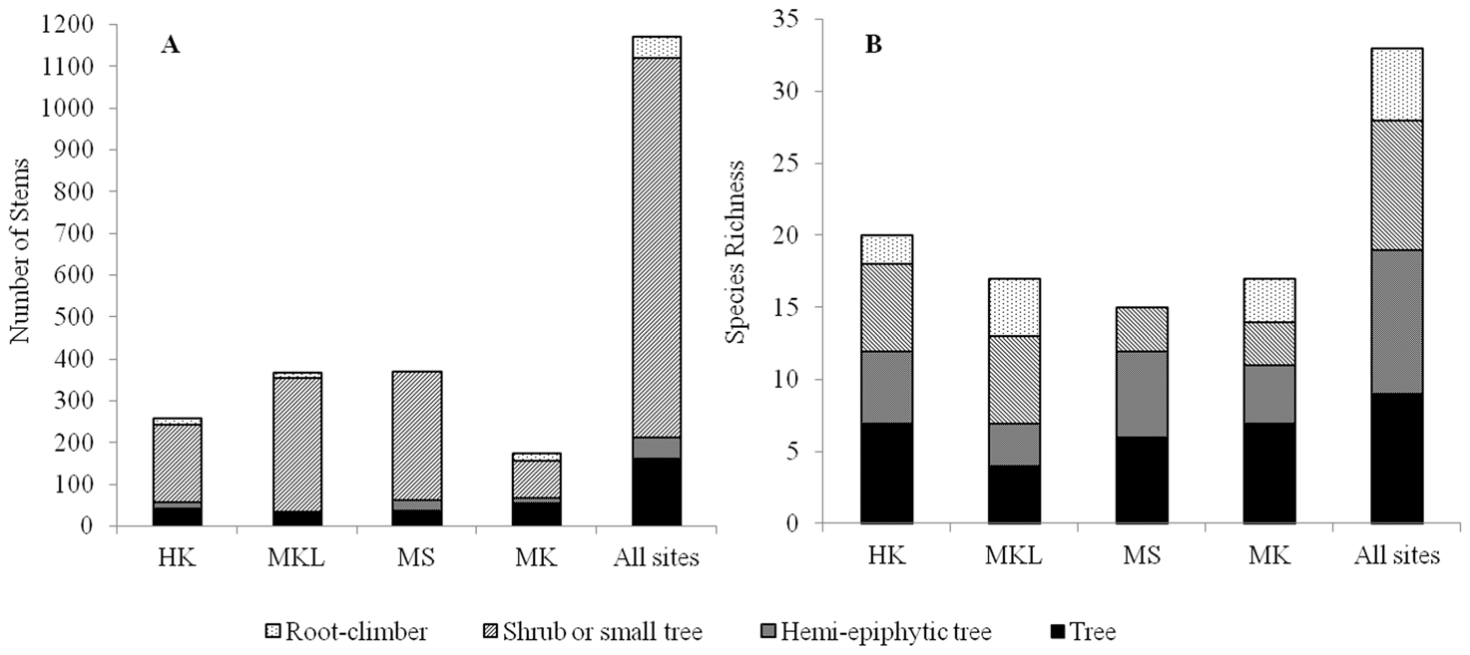
Stem abundance (A) and species richness (B) at four study sites: (HK) Huay Kaew stream, (MKL) Mae Klang stream, Mae Sa (MS) stream and Mae Ka stream (MK).

A total of 1169 individual fig trees were recorded in the surveys along the four streams with 174 (14.88%) individuals at MK, 258 (22.07%) at HK, 370 (31.65%) at MS and 367 (31.39%) at MKL. These *Ficus* communities are dominated by shrubs or small trees (Fig. 1A). Thirty-three fig tree species were recorded (Table S1 in File S3), representing 13 monoecious and 20 dioecious species from all six subgenera of *Ficus*. The largest taxonomic group was subgenus *Urostigma*, the strangler figs, with 10 species recorded. The second largest group consisted of eight species of free-standing trees (subgenus *Sycomorus*), followed by five species of creepers (subgenus *Synoecia*), with subgenera *Ficus* and *Sycidium* (both comprising small shrubs) each represented by four species, and free-standing *Pharmacosycea* by two species. Many species co-occurred at each site (17 species were present at MK, 20 at HK, 15 at MS and 17 at MKL) (Fig. 1B). Five species were common and occurred in all four sites including *F. auriculata* Lour, *F. hispida* L.f., *F.semicordata* Buch.-Ham. ex Sm., *F.squamosa* Roxb. and *F. microcarpa* L.f. Fourteen species were local and occurred at only one of four sites (Table S1 in File S3).

From 1–11 fig tree species were recorded per belt transect. The mean fig tree density across all belt transects was 292.25±94.45 stems/ha. The highest density was recorded for *F. squamosa* (117±81.95 stems/ha), followed by *F. ischnopoda* Miq. (68.25±89.88 stems/ha), *F. hirta* Vahl (23.75±39.30 stems/ha), *F. semicordata* (9±5.94 stems/ha), *F. auriculata* (7±2.16 stems/ha), *F. praetermissa* Corner (6.5±13.0 stems/ha), *F. fistulosa* Reinw. ex Blume (6.25±6.24 stems/ha), and *F. sagittata* J. Konig ex Vahl (5.25±4.57 stems/ha). The remaining species had densities of less than 5 stems/ha. One-way ANOVA showed that among study sites, there were no significant differences between numbers of fig trees and sites, or species richness and sites (Table 2).

**Table 2:** Descriptive statistics for diversity indices in the study sites.

Indices	Study site	Mean* (n = 10)	S.D.	Min.	Max.
Species Richness	Huay Kaew stream	4.30^a^	1.57	2.00	8.00
	Mae Klang stream	3.60^a^	1.65	1.00	7.00
	Mae Ka stream	5.70^a^	2.67	3.00	11.00
	Mae Sa stream	5.30^a^	2.71	3.00	11.00
Abundance	Huay Kaew stream	25.80^a^	13.74	8.00	49.00
	Mae Klang stream	36.70^a^	22.87	2.00	61.00
	Mae Ka stream	17.40^a^	11.22	7.00	41.00
	Mae Sa stream	37.00^a^	18.28	19.00	74.00
Shannon	Huay Kaew stream	1.00^ab^	0.50	0.12	1.88
	Mae Klang stream	0.72^a^	0.35	0.00	1.09
	Mae Ka stream	1.42^b^	0.46	0.96	2.04
	Mae Sa stream	0.97^ab^	0.31	0.54	1.43
Simpson	Huay Kaew stream	0.55^ab^	0.25	0.05	0.85
	Mae Klang stream	0.41^a^	0.20	0.00	0.60
	Mae Ka stream	0.75^b^	0.15	0.56	0.95
	Mae Sa stream	0.51^a^	0.12	0.29	0.66
Evenness	Huay Kaew stream	0.67^ab^	0.24	0.18	0.92
	Mae Klang stream	0.54^a^	0.22	0.00	0.76
	Mae Ka stream	0.86^b^	0.10	0.71	0.98
	Mae Sa stream	0.62^a^	0.09	0.49	0.72

*Letters (^a,b^) mean that different superscripts are significantly different (p<0.01) within the same column.

### Fig Tree Diversity

**Figure 2:**
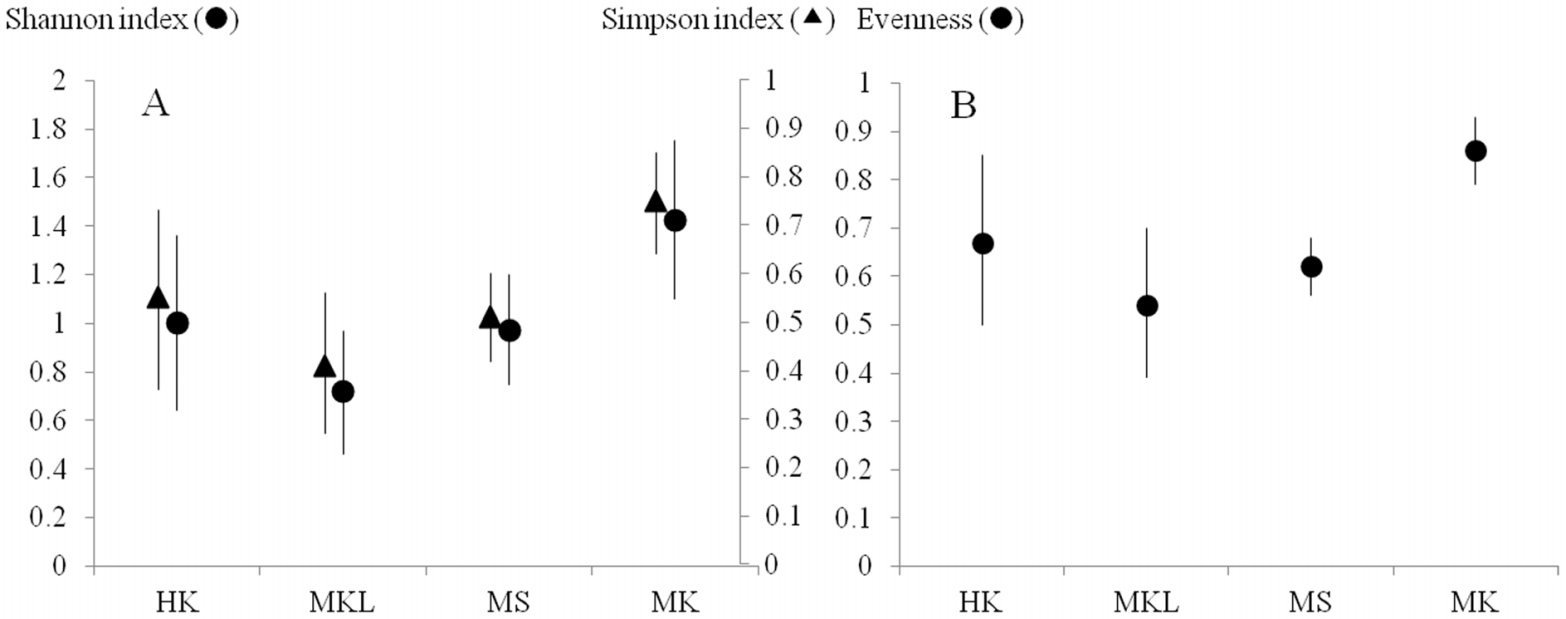
Estimates of diversity for riparian fig tree species at four study sites: (HK) Huay Kaew stream, (MKL) Mae Klang stream, MaeS (MS) stream and Mae Ka stream (MK). (A), mean diversity (±95% CI) for each site according to the Shannon’s index (circles) and Simpson index (triangles). (B), evenness at each study site is based on the Shannon’s index (circles); vertical lines indicate the 95% CI.

Despite an absence of significant differences in species numbers, there were significant differences in fig tree diversity across the four sites (Table 2), as measured by Shannon's index (H'), Evenness index (E) and Simpson’s diversity index (1-D) (one-way ANOVA, *F* = 5.039, *P*<0.001), *F* = 5.642, P<0.001 and *F* = 5.667, *P*<0.001, respectively) (Figs. 2A, 2B). Tukey HSD tests revealed that all diversity indices and evenness values for MK were significantly higher than at the other sites (all *P*≤0.01), whereas HK, MKL and MS did not differ significantly in their diversity indices (Table 2).

### Fig Tree Distributions

**Figure 3:**
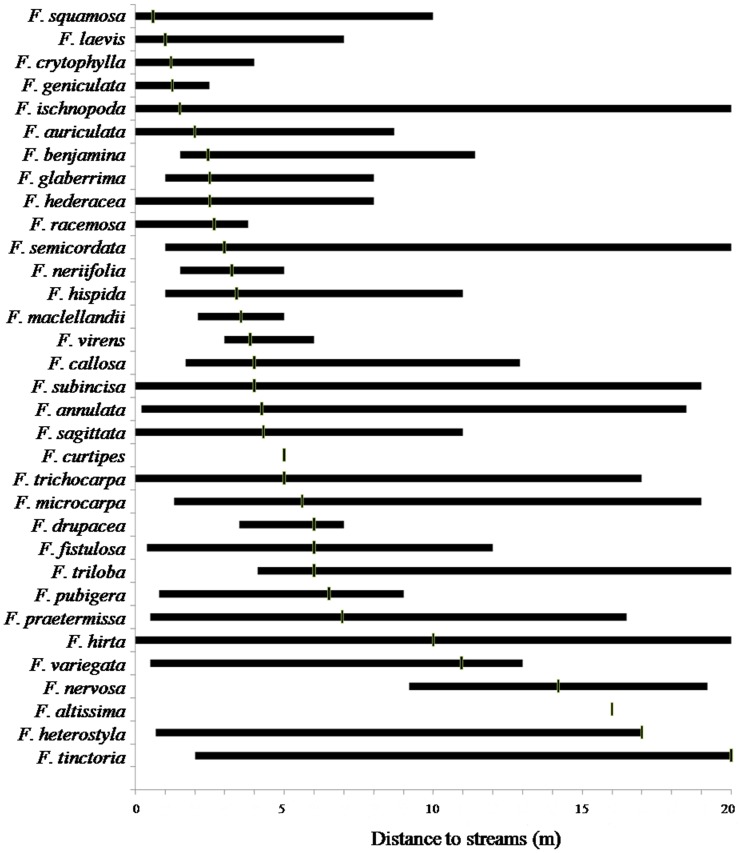
Median distance to streams (vertical solid lines) and ranges of occurrence of *Ficus* species (all four sites combined). See Table S2 in File S3 for number of stems of each species and where 75% of the total stems occur.

The median distance of occurrence of individual fig stems from stream edges varied between 0.6 and 20 m. *F. auriculata, F. ischnopoda, F. laevis*, *F. squamosa*, *F. geniculata* and *F. crytophylla* grew closest to the streams, with median distances to the streams of between 0.5 and 2 m (Fig. 3). Twenty one species occurred at a median distances between 2 and 8 m. and the six species that were least closely associated with the streams, with no stems within 10 m of the stream edges. were *F. nervosa*, *F. hirta*, *F. variegata*, *F. altissima*, *F. heterostyla* and *F. tinctoria*.

In terms of percentiles of the total distributions of stems, fifty percent of *F. squamosa* were within 0.6m from the streams. Twelve species had seventy-five percent of their stems at distances less than 5 m away from the streams (*F. ischnopoda*, *F. nerrifolia*, *F. hederacea, F. laevis, F. crytophylla, F. auriculata, F. racemosa, F. geniculata, F. benjamina, F. glaberrima, F. maclellandii* and *F. curtipes*) (Table S2 in File S3).

Across sites, fig species richness varied with distance from the streams, with nearest the stream (0–5 m) having the highest number of species and the farthest distance (≥15–20 m) the lowest number (Table 3). The same pattern was evident for stem abundance. A two-way ANOVA showed that there was no significant difference between streams or sites (*F*_3,144_ = 2.808, *P* = 0.042) in the distributions of stems from the streams. There was also no significant difference in fig species richness distances between sites (*F*_3,144_ = 1.153, *P* = 0.330). However, the mean numbers of stems in the different distance bands were significantly different within sites (*F*_3,144_ = 57.210, *P*<0.001), as were species richness (*F*_3,144_ = 54.894, *P*<0.001) (Table 4). One-way ANOVA showed that the first distance bands (0–5 m) varied highly significantly in terms of stem abundance (HK; *F*_3,36_ = 4.088, *P*<0.05, MKL; *F*_3,36_ = 13.76, *P*<0.001, MS; *F*_3,36_ = 42.34, *P*<0.001, MK; *F*_3,36_ = 15.43, *P*<0.001). When compared between sites, MS had significantly (*P*<0.05) greater number of stems in the streamside zone (0–5 m) than HK, MKL and MS (Tukey tests).

**Table 3:** Differences in fig tree mean species richness and density in different river distance bands (four study sites combined).

Distance stratafrom streams	Total speciesrichness	Number ofindividuals	Species richness(Mean ± SD)	Density (stems/ha)(Mean ± SD)
1. (0–5 m)	24	937	4.100±2.17	234.25±114.81
2. (≥5–10 m)	17	128	1.375±1.75	32.00±13.49
3. (≥10–15 m)	11	43	0.525±0.78	10.75±8.26
4. (≥15–20 m)	8	61	0.350±0.74	15.25±24.62

**Table 4:** One-way ANOVA for number of stems, species richness and diversity between three elevation ranges of each quadrats (All four sites combined, n = 40).

	MS	*F*-Value	*p*
Number of stems	2118.1	8.731	0.0007*
Richness	4.306	1.098	0.344
Shannon (H′)	0.618	3.007	0.062
Simpson	0.150	3.298	0.048
Evenness	0.160	4.162	0.023
Density	0.002	8.69	0.0008*

Asterisks* indicates significant differences.

**Figure 4:**
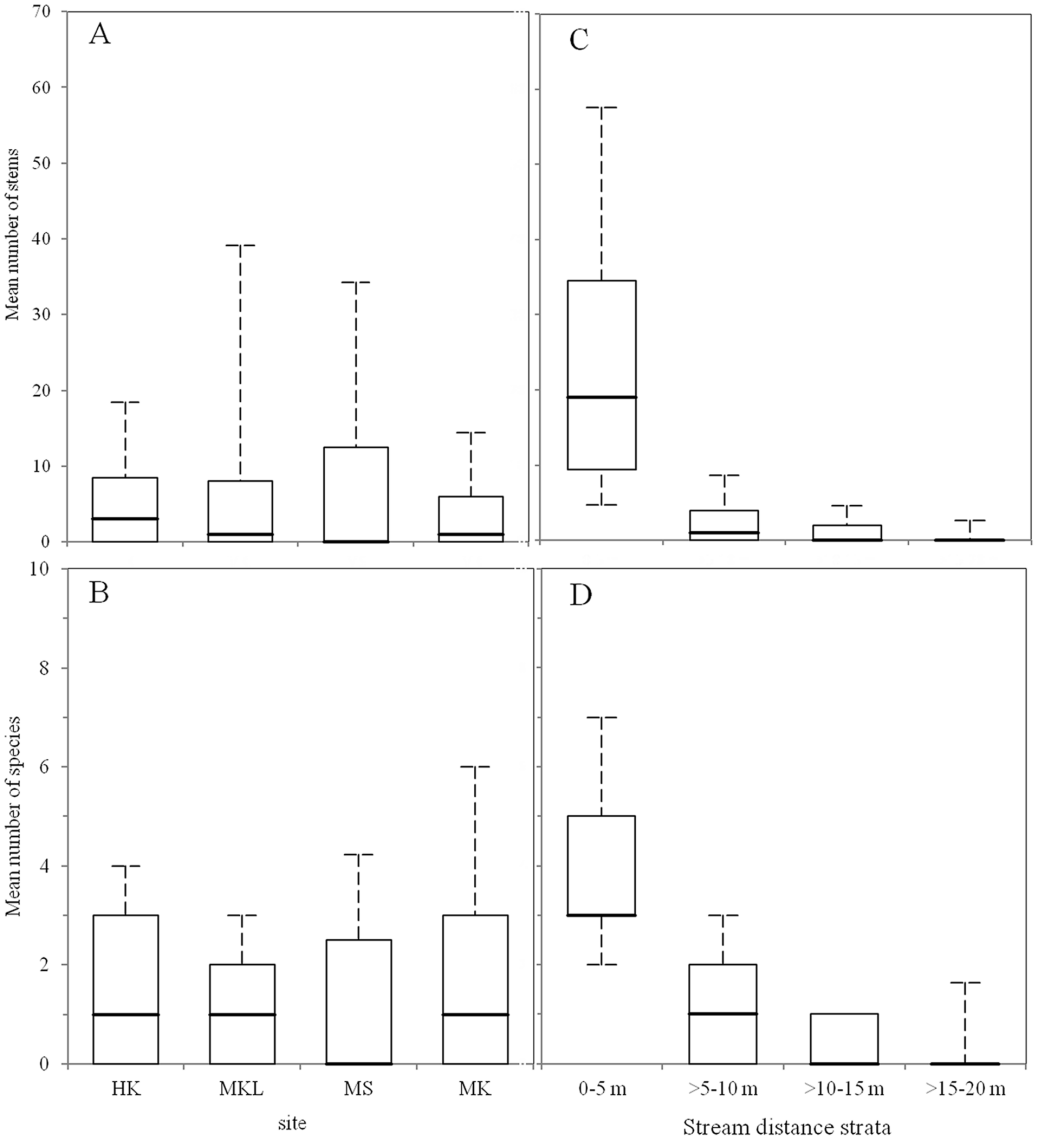
Mean number of stems (A, C) and mean number of species (B, D) in four stream sites (left) and at each stream distance stratum (right). The boxplots describe the relationship between mean number of stems and species richness in each stream and stream distance strata. The maximum and minimum extents of the boxes indicate 25th and 75th percentiles and thick solid lines indicate the medians.

Comparisons of the box plots suggest that there was no significant difference between the numbers of stems (*F* = 2.808, *P* = 0.042) and species (*F* = 1.1.53, *P* = 0.330) between sites (Fig. 4A, 4B), but there appeared to be differences between sites and highly significant relationships with distance to the stream in terms of both the number of stems (*F* = 57.210, *P*<0.001) (Fig. 4C) and species richness (*F* = 54.894, *P*<0.001) (Fig. 4D). Two-way ANOVA showed that there was a significant interaction effect between site and stream distance strata (*F*_9,144_ = 4.831, *P*<0.001) on the mean number of stems and no significant difference between mean number of species (*F* = 1.1.68, *P* = 0.320).

Within the forty quadrats, there was a significant relationship between species richness (*r* ^2^ = 0.421, *P*<0.001) and distance from the streams. In addition, there was a significant positive relationship between number of stems (*r* ^2^ = 0.311, *P*<0.001) and stem density (*r* ^2^ = 0.308, *P*<0.001) and proximity to streams, with the overall number of stems increasing with proximity to the streams.

### Fig Composition Along An Elevation Gradient

Fig tree abundance and density varied significantly between elevation ranges and forest types (Table 4). Tukey’s tests indicated that the elevation range 600–1000 m. with mixed deciduous and evergreen forest types had significantly higher densities of fig trees than the others (*F* = 8.89, *P*<0.001). In contrast, there were no statistically significant differences in species richness and diversity between the elevation ranges (Table 4). Linear regression analysis showed that there was also no relationship between mean elevation of quadrats and number of stems, species richness, or diversity (all values *r* ^2^<0.08, *P*>0.04).

**Figure 5:**
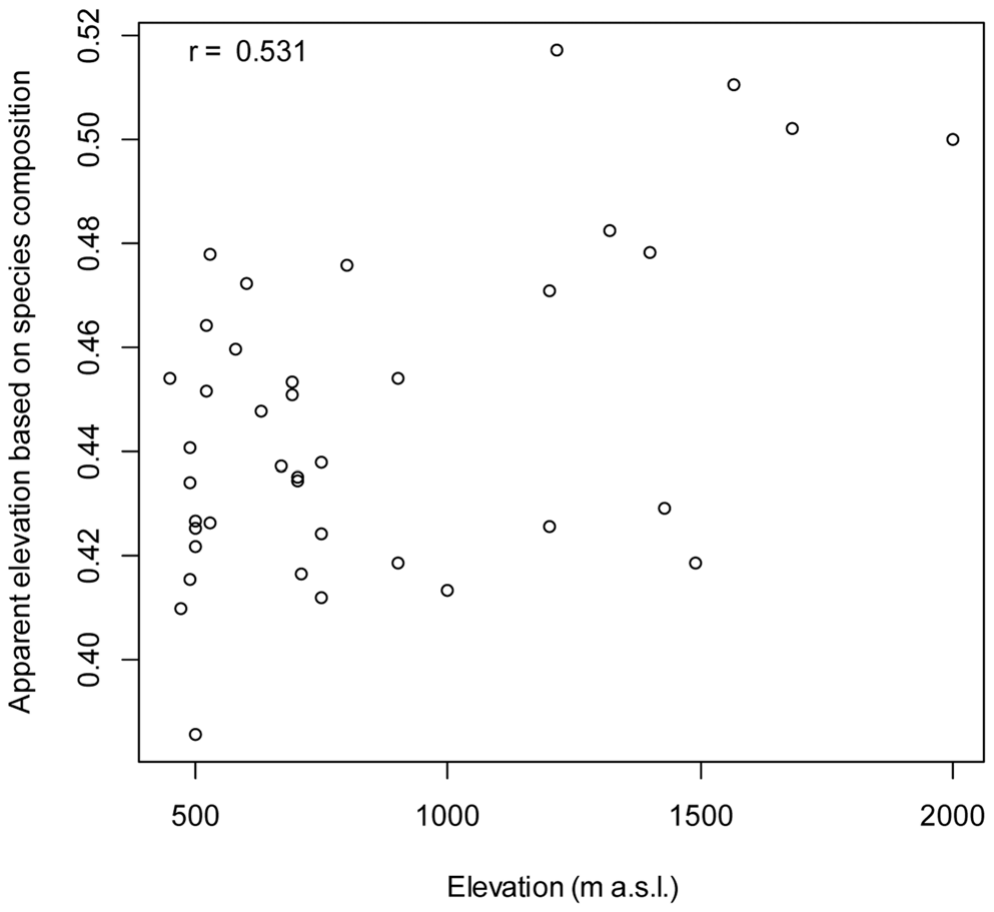
Plot of ordination analysis (Fuzzy Set Ordination; FSO) based on binary data, (presence/absence of species) and mean elevation in each quadrat. Sørensen indices were evaluated with rank and linear correlations with the apparent elevation gradient.

In the analysis of community composition using fuzzy set ordination (FSO) with binary (presence/absence) data (Fig. 5), we found a significant relationship between species abundance and composition as a function of elevation range (*F* = 2.118, *P*<0.001). The FSO indicated that there was a relationship between species composition of quadrats and elevation ranges (*r* ^2^ = 0.263, *P*<0.001). The relationship between elevation and”apparent elevation based on species composition” was moderate, with a correlation of 0.531, meaning that we can predict the elevation range to some degree of a site based on its species composition. At elevations lower than 1,000 m asl. we observed greater differences among quadrats in species composition (more turnover) than at higher elevations, where riparian fig tree species richness is more limited.

## Discussion

High species richness is a frequently cited property of riparian zones [30], [32], [35], [59] and may be related to factors including disturbance [60], [61], productivity, flow-facilitated dispersal of propagules [62], and the diversity of physical conditions present at the interface between aquatic and terrestrial ecosystems [30]. Fig trees are commonly found in moderate to moist areas near rivers or streams [63], [64] and riparian zones in northern Thailand were found to be extremely rich in *Ficus* species. We recorded 33 species in 40 plots that had a total area of only about 4 ha. This is about two thirds of all the *Ficus* species native to Northern Thailand [23] and more than one quarter of all native fig tree species in Thailand [51]. This high level of species richness is by no means unusual for fig trees and is comparable to other sites in the region [65]. Elsewhere in Thailand, 50 percent of the 38 *Ficus* taxa recognized by [8] were recorded at least occasionally from riparian situations, and several species were only recorded from this habitat. Similarly, Kong-ied [43] reported that *Ficus* L. was the most diverse genus in the riparian forest along the Sok Canal, Surat Thani Province, southern Thailand, though only 13 species were represented. The fig trees were also abundant at our study sites and these results emphasize that, as in some other habitats, riparian *Ficus* are likely to be keystone resources for threatened frugivores such as hornbills [66] as well as for many other species of birds and mammals.

Shrubs, creepers, stranglers and free-standing trees were all present in the riparian vegetation. A small number of the *Ficus* species were extreme riverine habitat specialists capable of growing on rocks in mid-stream (rheophytes), some other species were bank-side specialists largely restricted to riparian zones, and others were more widespread species that are also found routinely in other habitats [10]. In this study, the most common *Ficus* species at the four sites, and the species most closely located to the streams, was *F. squamosa*. It is a rheophytic shrub with rooting stolon-like stems, and is particularly associated with fast-flowing streams. Our field observations found that this species is tolerant of extreme disturbance during rainy season flooding events. It also displays anatomical adaptations for seed dispersal by water [10]. In addition to *F. squamosa, F. auriculata, F. geniculata, F. ischnopoda,* and *F.crytophylla* all had median distances to stream edges of less than 2 m. Environmental factors related to proximity to streams, such as disturbance, readily available water and increased light from the open canopy present above are likely to have been influencing their distributions [39]. Hemi-epiphytic strangler figs were found mostly at greater distances from the streams and their association with the riparian zone appears to be more casual. They germinate from seeds deposited in the canopy and are likely to be less responsive to the increased light levels at stream edges, and because they are epiphytic as young plants they also display adaptations for resistance to dehydration, so the elevated soil moisture near streams will be less important [11]. Consequently, the availability of suitable host trees may be more important than where the host trees are growing.

There was significant positive relationships between species richness, abundance and density of figs with proximity to streams. Higher light levels and the availability of water clearly play the important role in organizing the distribution of riparian figs. The relationship between hydrology and riparian plant composition has previously been identified as an important research area [59], [61] that requires further interdisciplinary research.

Ecological studies of riparian zones have been predominantly in North America. Temperate research from there and elsewhere has demonstrated the importance of the disturbance caused by flood scour events for the maintenance of local biodiversity [67]. Community processes in tropical riparian zones are less well understood, but they are likely to show significant differences from temperate examples. Hydrological regimes may or may not be more highly variable in the tropics, depending on local rainfall regimes, and predictable seasonal events such as snow melt will usually be absent. The chemical composition of stream substrates, and of adjacent land, may also often be different. Tropical riparian zones also have a different plant and animal taxonomic composition and much higher species richness. Interactions between plants and animals may also be more significant, with greater dependence on animal pollination and seed dispersal. Chantarasuwan *et al*. [68] concluded that suitable habitats for riparian fig species was determined mainly by moisture, with aspect and slope of less importance. In South America, Banack [69] found that *F. insipida* occurs only along larger streams and rivers and concluded that it is more establishment-limited than disperser-limited, despite being dispersed mainly by bats and fish.

*Ficus* species richness and abundance (numbers of stems) did not differ between the four streams we examined. The species diversity of the Mae Ka stream (MK) was nonetheless higher than those of the others. It was situated at a lower elevation and had a different substrate to the other streams (with a combination of limestone bed rock and granite). This finding is consistent with that of Munishi [70], who revealed that there was difference in species richness, density and diversity of tree resources outside forest in river strata on the Southern side of Mount Kilimanjaro. This variation did not follow any particular pattern with regard to distances from river catchments. Topography and soil types are often important factors in the distribution of tree species [71] and the nature of the dipterocarp forest at the MK sites may also have had an influence on fig tree distribution.

There were more species and a higher density of fig trees between 400–600 m asl. The distribution of species richness along elevation gradients is governed by a series of interacting biological, climatic and historical factors [72] and elevation represents a complex gradient along which many environmental variables change simultaneously [73]. Other factors, such as soil fertility and topography may also affect the patterns of species richness along elevation gradients. In mountain regions, the pattern of different forest types and other communities often corresponds to elevation and topography. Variation in microclimate with topography and elevation is also a major factor of species distribution within a forest landscape [74]. While Harrison *et al*. [75] found that most hemi-epiphytic fig trees are widely distributed, the assemblages differed substantially between sites. They suggested that large-scale habitat associations may also influence fig tree species richness at higher elevations.

In conclusion, tropical riparian vegetation influences several important ecological functions such as stream bank stabilization, reduction of flood velocities, shading and the provision of food for animals. It is also a particularly vulnerable and threatened habitat in Thailand and elsewhere [30], [32]–[38]. We found that in northern Thailand fig trees are a numerically important and diverse component of riparian vegetation, and that this habitat supports a high proportion of all figs in the region, comprising a mix of growth forms and both habitat specialists and generalists. Streams on limestone appear to differ in character to those on acid substrates, and there is some variation in community composition with altitude. This study emphasizes the likely conservation importance of fig trees in riparian areas of Thailand, but their role in broader ecosystem functioning remains largely unknown.

## Supporting Information

Figure S1 **The locations of four riparian fig tree study sites in Chiang Mai Province, Northern Thailand.** 1. Mae Ka stream (MK) 2. Mae Sa stream (MS) 3. Huay Kaew stream (HK) 4. Mae Klang stream (MKL).
(TIF)

Figure S2 **Distribution of plot sites along an elevation gradient followed the forest classification scheme in Thailand; dry deciduous dipterocarp, mixed diciduous and evergreen, and hill evergreen respectively.** The elevation gradient was divided into three ranges i.e. 400–600 m asl (n = 16 plots), 600–1,000 m asl. (n = 11 plots) and the final one at >1,000 m asl. (n = 13 plots).
(TIF)

File S1 **Tables S1 and S2.** Table S1. Species list of riparian figs present along four streams in Chiang Mai, Northern Thailand. Table S2. Median distances to streams of *Ficus* species (all four sites combined). (N = total stems, Median = median distance to stream, SD = standard deviation, p75 = distance from streams at which 75% of stems occur, min = minimum distance, and max = maximum distance recorded from stream edges).
(DOCX)
