# Supplementary figures and images for: Riparian Ficus Tree Communities: The Distribution and Abundance of Riparian Fig Trees in Northern Thailand
Source: PLoS One. 2014 Oct 13;9(10):e108945. doi: 10.1371/journal.pone.0108945 (PMC4195654; doi:10.1371/journal.pone.0108945)

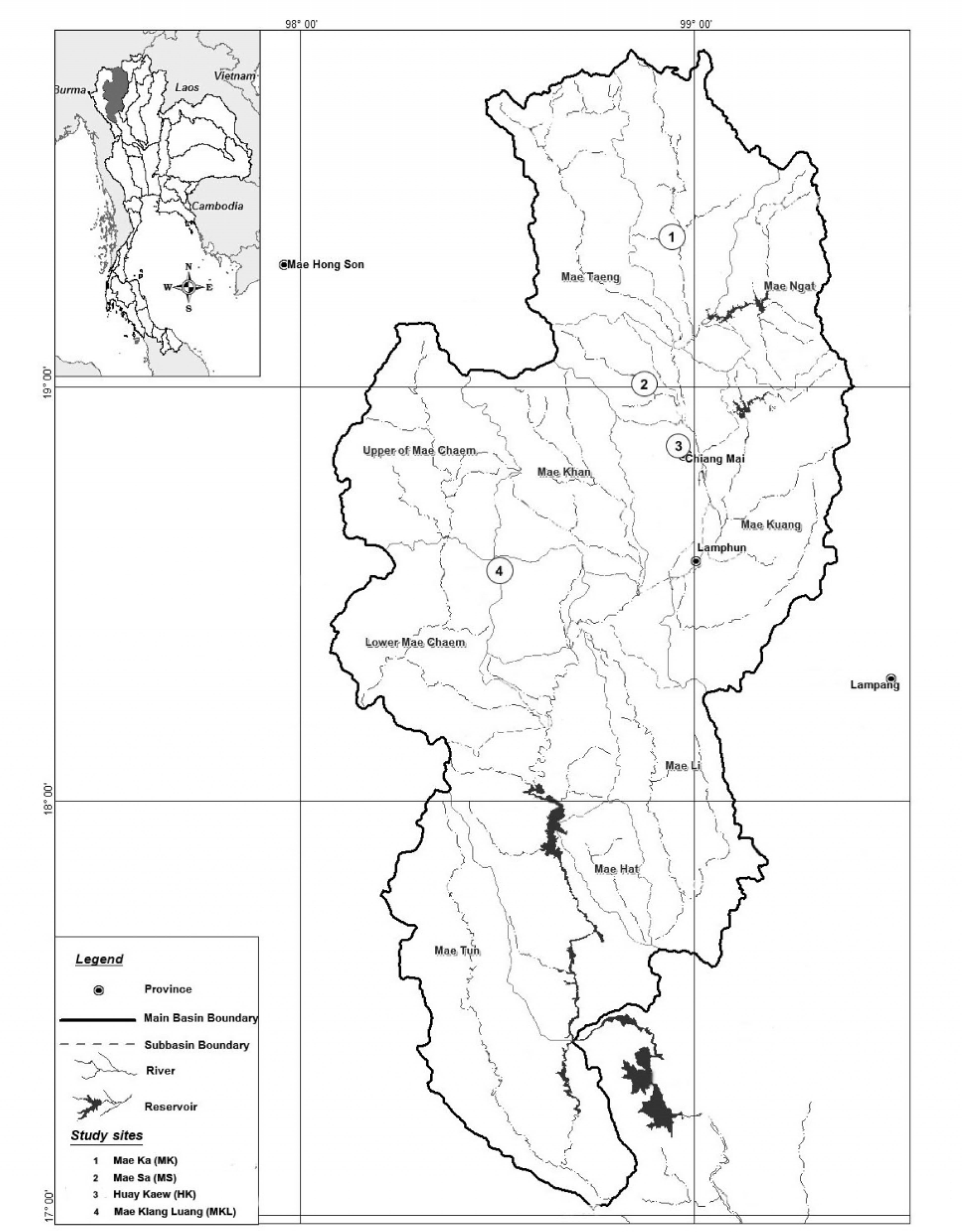

Supplement: Figure S1 — The locations of four riparian fig tree study sites in Chiang Mai Province, Northern Thailand. 1. Mae Ka stream (MK) 2. Mae Sa stream (MS) 3. Huay Kaew stream (HK) 4. Mae Klang stream (MKL). (TIF) [file pone.0108945.s001.tif]

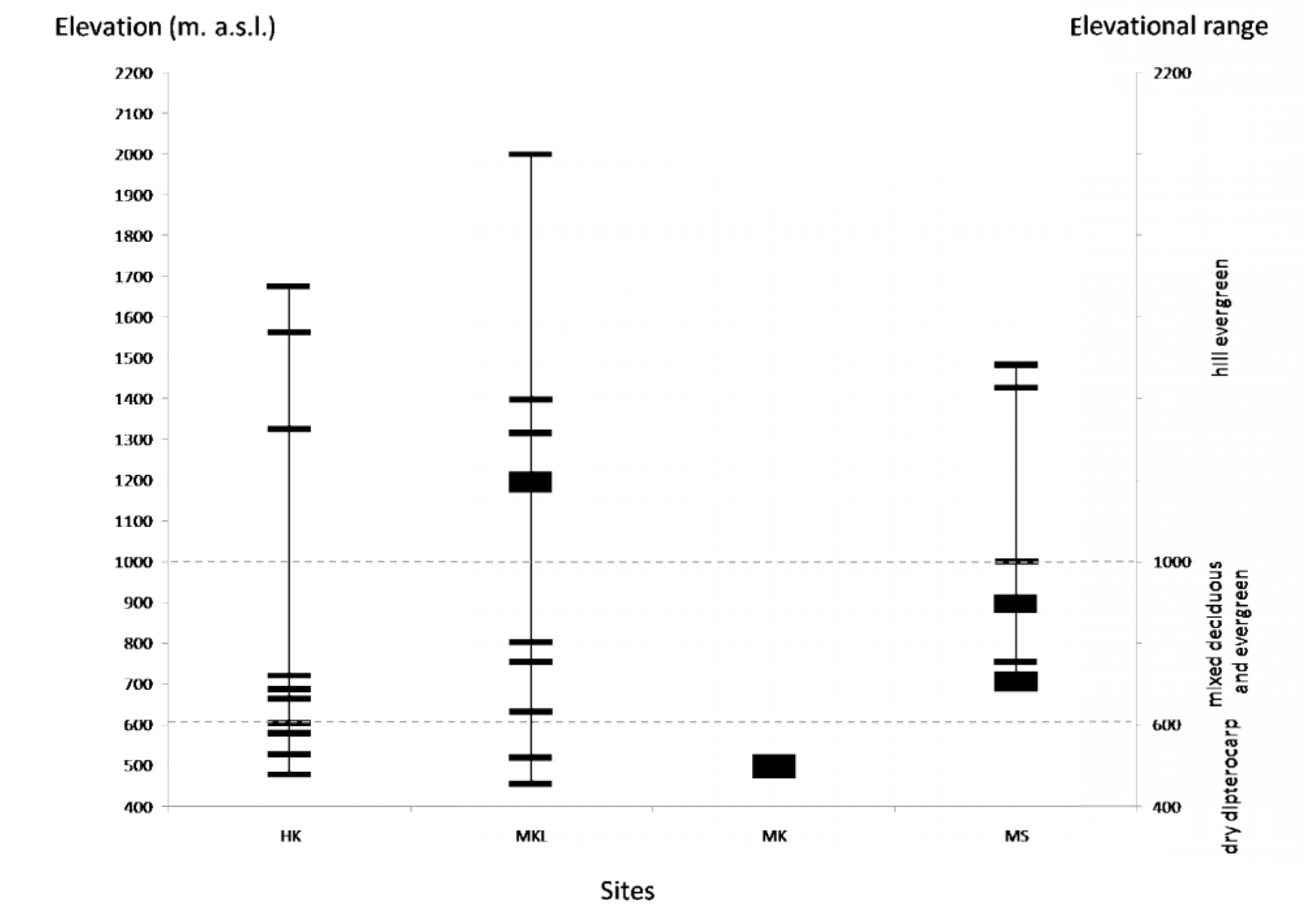

Supplement: Figure S2 — Distribution of plot sites along an elevation gradient followed the forest classification scheme in Thailand; dry deciduous dipterocarp, mixed diciduous and evergreen, and hill evergreen respectively. The elevation gradient was divided into three ranges i.e. 400–600 m asl (n = 16 plots), 600–1,000 m asl. (n = 11 plots) and the final one at >1,000 m asl. (n = 13 plots). (TIF) [file pone.0108945.s002.tif]
