# Supplementary material for: Riparian Ficus Tree Communities: The Distribution and Abundance of Riparian Fig Trees in Northern Thailand
Source: PLoS One. 2014 Oct 13;9(10):e108945. doi: 10.1371/journal.pone.0108945 (PMC4195654; doi:10.1371/journal.pone.0108945)
Supplement: File S1 — Tables S1 and S2. Table S1. Species list of riparian figs present along four streams in Chiang Mai, Northern Thailand. Table S2. Median distances to streams of Ficus species (all four sites combined). (N = total stems, Median = median distance to stream, SD = standard deviation, p75 = distance from streams at which 75% of stems occur, min = minimum distance, and max = maximum distance recorded from stream edges). (DOCX) [file pone.0108945.s003.docx]

**Table S1.** Species list of riparian figs present along four streams in Chiang Mai, Northern Thailand.

|  | **Subgenus** | **Species** | **Habit** | **Establishment**  **sites** | **Number**  **of**  **individuals** | **Number of individuals**  **at each study site** | | | |
| --- | --- | --- | --- | --- | --- | --- | --- | --- | --- |
|  |  |  |  |  |  | **Granite** | | | **Limestone** |
|  |  |  |  |  |  | **HK** | **MKL** | **MS** | **MK** |
| Dioecious | *Ficus* | *Ficus ischnopoda* Miq. | rheophytic shrub and small tree | soil and rock | 273 | 7 | 194 | 72 |  |
|  |  | *Ficus hirta* Vahl | shrub and small tree | soil | 95 | 83 | 12 |  |  |
|  |  | *Ficus triloba* Buch.-Ham. *ex* Wall. | Tree | soil | 5 | 5 |  |  |  |
|  |  | *Ficus neriifolia* Sm. | shrub or tree | soil | 2 |  | 2 |  |  |
|  | *Synoecia* | *Ficus sagittata* J. Konig *ex* Vahl | root-climber | soil | 21 | 10 | 3 |  | 8 |
|  |  | *Ficus hederacea* Roxb. | root-climber | soil | 16 |  | 6 |  | 10 |
|  |  | *Ficus laevis* Blume | root-climber | soil | 8 | 7 | 1 |  |  |
|  |  | *Ficus trichocarpa* Blume. | scandent shrub | soil | 7 |  | 7 |  |  |
|  |  | *Ficus pubigera* (Miq.) Wall. *ex* Brandis | root-climber | soil | 4 |  | 4 |  |  |
|  | *Sycidium* | *Ficus praetermissa* Corner | creeping shrub | soil | 26 |  |  |  | 26 |
|  |  | *Ficus subincisa* J.E. Smith | shrub and small tree | soil | 15 | 8 | 2 |  | 5 |
|  |  | *Ficus tinctoria* G. Forster | shrub and small tree | soil | 7 | 1 |  | 2 | 4 |
|  |  | *Ficus crytophylla* (Wall. *ex* Miq.) Miq. | shrub or treelet | soil | 13 | 13 |  |  |  |
|  | *Sycomorus* | *Ficus auriculata* Lour. | tree | soil | 30 | 4 | 8 | 7 | 11 |
|  |  | *Ficus fistulosa* Reinw. *ex* Blume | tree | soil | 25 | 13 | 2 |  | 10 |
|  |  | *Ficus hispida* L.f. | tree | soil | 16 | 3 | 1 | 11 | 1 |
|  |  | *Ficus semicordata* Buch.-Ham. *ex* Sm. | tree | soil | 36 | 4 | 17 | 5 | 10 |
|  |  | *Ficus variegata* Blume | tree | soil | 12 |  |  | 1 | 11 |
|  |  | *Ficus squamosa* Roxb. | rheophytic shrub | rock | 468 | 74 | 104 | 236 | 54 |
|  |  | *Ficus heterostyla* Merr. | shrub or tree | soil | 11 | 11 |  |  |  |
| Monoecious | *Pharmacosycea* | *Ficus callosa* Willd. | tree | soil | 12 | 2 |  | 1 | 9 |
|  |  | *Ficus nervosa* Heyne *ex* Roth | root-climber | soil | 2 |  |  |  | 2 |
|  | *Sycomorus* | *Ficus racemosa* L. | tree | soil | 14 |  |  | 12 | 2 |
|  | *Urostigma* | *Ficus annulata* Blume | hemi-epiphytic, tree | host tree | 10 |  |  | 2 | 8 |
|  |  | *Ficus geniculata* Kurz | hemi-epiphytic, tree | host tree | 2 |  |  | 2 |  |
|  |  | *Ficus virens* Aiton | hemi-epiphytic, tree | host tree | 4 |  |  | 4 |  |
|  |  | *Ficus benjamina* L. | hemi-epiphytic, tree | host tree | 6 | 6 |  |  |  |
|  |  | *Ficus drupacea* Thunb. | hemi-epiphytic, tree | host tree | 3 |  |  | 3 |  |
|  |  | *Ficus glaberrima* Blume | hemi-epiphytic, tree | host tree | 9 | 2 | 1 | 6 |  |
|  |  | *Ficus maclellandii* King | hemi-epiphytic, tree | host tree | 2 |  | 1 |  | 1 |
|  |  | *Ficus microcarpa* L.f. | hemi-epiphytic, tree | host tree | 13 | 3 | 2 | 6 | 2 |
|  |  | *Ficus curtipes* Corner | hemi-epiphytic, tree | host tree | 1 | 1 |  |  |  |
|  |  | *Ficus altissima* Blume | hemi-epiphytic, tree | host tree | 1 | 1 |  |  |  |

**Table S2.** Median distances to streams of *Ficus* species (all four sites combined). (N = total stems, Median = median distance to stream, SD = standard deviation, p75 = distance from streams at which 75% of stems occur, min = minimum distance, and max = maximum distance recorded from stream edges)

| **Species** | | **N** | **Median** | **SD** | | **p75** | **Min**  **(m)** | | **Max**  **(m)** |
| --- | --- | --- | --- | --- | --- | --- | --- | --- | --- |
| *Ficus squamosa* Roxb. | 468 | 0.6 | 1.5 | 1.4 | | | 0.0 | 10.0 |  |
| *Ficus laevis* Blume | 8 | 1.0 | 2.5 | 3.1 | | | 0.0 | 7.0 |  |
| *Ficus crytophylla* (Wall. *ex* Miq.) Miq. | 13 | 1.2 | 1.0 | 1.5 | | | 0.0 | 4.0 |  |
| *Ficus geniculata* Kurz | 2 | 1.3 | 1.8 | 1.9 | | | 0.0 | 2.5 |  |
| *Ficus ischnopoda* Miq. | 273 | 1.5 | 2.4 | 2.6 | | | 0.0 | 20.0 |  |
| *Ficus auriculata* Lour. | 28 | 2.0 | 2.3 | 3.1 | | | 0.0 | 8.7 |  |
| *Ficus benjamina* L. | 6 | 2.5 | 3.7 | 3.6 | | | 1.5 | 11.4 |  |
| *Ficus glaberrima* Blume | 9 | 2.5 | 2.3 | 3.6 | | | 1.0 | 8.0 |  |
| *Ficus hederacea* Roxb. | 16 | 2.5 | 2.2 | 3.5 | | | 0.0 | 8.0 |  |
| *Ficus racemosa* L. | 14 | 2.7 | 1.2 | 3.0 | | | 0.0 | 3.8 |  |
| *Ficus semicordata* Buch.-Ham. *ex* Sm. | 36 | 3.0 | 4.3 | 5.7 | | | 1.0 | 20.0 |  |
| *Ficus neriifolia* Sm. | 2 | 3.3 | 2.5 | 4.1 | | | 1.5 | 5.0 |  |
| *Ficus hispida* L.f. | 16 | 3.4 | 3.1 | 7.1 | | | 1.0 | 11.0 |  |
| *Ficus maclellandii* King | 2 | 3.6 | 2.1 | 4.3 | | | 2.1 | 5.0 |  |
| *Ficus virens* Aiton | 4 | 3.9 | 1.5 | 4.8 | | | 3.0 | 6.0 |  |
| *Ficus callosa* Willd. | 12 | 4.0 | 3.1 | 5.4 | | | 1.7 | 12.9 |  |
| *Ficus subincisa* J.E. Smith | 15 | 4.0 | 4.9 | 6.2 | | | 0.0 | 19.0 |  |
| *Ficus annulata* Blume | 12 | 4.3 | 6.0 | 11.2 | | | 0.2 | 18.5 |  |
| *Ficus sagittata* J. Konig *ex* Vahl | 21 | 4.3 | 3.2 | 6.0 | | | 0.0 | 11.0 |  |
| *Ficus curtipes* Corner | 1 | 5.0 | 0.0 | 0.0 | | | 5.0 | 5.0 |  |
| *Ficus trichocarpa* Blume. | 7 | 5.0 | 7.0 | 13.5 | | | 0.0 | 17.0 |  |
| *Ficus microcarpa* L.f. | 13 | 5.6 | 5.4 | 8.0 | | | 1.3 | 19.0 |  |
| *Ficus drupacea* Thunb. | 3 | 6.0 | 1.8 | 6.5 | | | 3.5 | 7.0 |  |
| *Ficus fistulosa* Reinw. *ex* Blume | 25 | 6.0 | 3.7 | 8.7 | | | 0.4 | 12.0 |  |
| *Ficus triloba* Buch.-Ham. *ex* Wall. | 5 | 6.0 | 6.4 | 10.0 | | | 4.1 | 20.0 |  |
| *Ficus pubigera* (Miq.) Wall. *ex* Brandis | 4 | 6.5 | 3.6 | 7.9 | | | 0.8 | 9.0 |  |
| *Ficus praetermissa* Corner | 26 | 7.0 | 4.0 | 10.0 | | | 0.5 | 16.5 |  |
| *Ficus hirta* Vahl | 95 | 10.0 | 6.7 | 17.9 | | | 0.0 | 20.0 |  |
| *Ficus variegata* Blume | 12 | 11.0 | 4.9 | 12.0 | | | 0.5 | 13.0 |  |
| *Ficus nervosa* Heyne *ex* Roth | 2 | 14.2 | 7.1 | 16.7 | | | 9.2 | 19.2 |  |
| *Ficus altissima* Blume | 1 | 16.0 | 0.0 | 0.0 | | | 16.0 | 16.0 |  |
| *Ficus heterostyla* Merr. | 11 | 17.0 | 7.6 | 17.5 | | | 0.7 | 17.0 |  |
| *Ficus tinctoria* G. Forster | 7 | 20.0 | 9.3 | 20.0 | | | 2.0 | 20.0 |  |

**Figure S1.** The locations of four perennial streams riparian fig tree study sites in Chiang Mai Province, Northern Thailand. 1. Mae ka stream (MK) 2. Mae Sa stream (MS) 3. Huay Kaew stream (HK) 4. Mae Klang stream (MKL)

**Figure S2.** Distribution of plot sites along an elevation gradient followed the forest classification schemes in Thailand; dry deciduous dipterocarp, mixed diciduous and evergreen, and hill evergreen respectively. The elevation gradient was divided into three ranges i.e. 400-600 m asl (n = 16 plots), 600-1,000 m asl. (n = 11 plots) and the final one at >1,000 m asl. (n = 13 plots).
